# What seems to matter in public policy and the health of informal caregivers? A cross-sectional study in 12 European countries

**DOI:** 10.1371/journal.pone.0194232

**Published:** 2018-03-08

**Authors:** Laia Calvó-Perxas, Joan Vilalta-Franch, Howard Litwin, Oriol Turró-Garriga, Pedro Mira, Josep Garre-Olmo

**Affiliations:** 1 Girona Biomedical Research Institute (IdIBGI), Girona, Catalonia, Spain; 2 Memory and Dementia Assessment Unit, Hospital Santa Caterina, Institut d’Assistència Sanitària, Salt, Catalonia, Spain; 3 Department of Medical Sciences, University of Girona, Girona, Catalonia, Spain; 4 Paul Baerwald School of Social Work and Social Welfare, The Hebrew University in Jerusalem, Jerusalem, Israel; 5 Centro de Estudios Monetarios y Financieros, Banco de España, Madrid, Spain; Taipei Veterans General Hospital, TAIWAN

## Abstract

In Europe, informal caregiving is frequent and is expected to grow. Caregiving has an impact on caregivers’ health, but its effect may vary according to the policies of support that are available to caregivers. The aim of this study was to assess the association between the policies of support to caregivers available in 12 European countries and the health of caregivers, considering separately the policies based on financial help and those based on training and other non- financial services. We used data from 13,507 caregivers from 12 European countries from the fifth wave of the Survey of Health, Ageing and Retirement in Europe (SHARE) to build a path model. Poor health among caregivers was associated with living in a family-based care country (β = 0.50; 95% CI = 0.42–0.59), and with an increased extent of caregiving (β = 0.18; 95% CI = 0.15–0.22). Non-financial support measures seem to have a larger protective impact (β = –0.33; 95% CI = –0.38 - –0.28) on the health of caregivers than do financial support measures (β = 0.03; 95% CI = 0.01–0.04), regardless of the gender of the caregiver. According to our results, the currently available policies of support associated with better health among caregivers are those that: 1) provide them with some free time, 2) help them to deal emotionally with caregiving, and 3) give them skills to both improve the care situation and to deal with it better.

## Introduction

The ageing of the population brings an increasing demand for public long-term care services, challenging the healthcare systems in Europe [1,2]. Informal caregivers may relieve this pressure, and many governments rely on them to provide support in times of welfare state retrenchment and cutbacks [3]. The number of informal caregivers around the world is already large, and recent research suggests that the number of people taking up the caregiver role will increase in the next years [4].

It is widely accepted that caregiving has an impact on caregivers’ health [5–7], and most caregivers have to deal with their own chronic illnesses as well [8,9]. Support measures are thus necessary to keep caregivers in good health, to maintain their quality of life, and to keep costs down, so that the informal caregiving system is maintained [10,11].

According to the Stress Process Model [12] and the Appraisal model [13] there are a number of interrelated determinants of stress among caregivers. These determinants have been categorized as contextual factors (i.e. demographic variables), moderators (i.e. psychosocial support, caregiver’s skills and personality) and stressors (i.e. financial strain, care situation), and they have been seen to exacerbate or ameliorate the emotional and health consequences of caregiving [14]. There is also a subjective appraisal of caregiving, which can modify the quality of life and the burden of the caregiver [13], which, in turn, can modify the health of the caregivers [15].

Caregiving is also shaped by the social context of each country, especially because the perceived duty to care for relatives varies across countries [16]. It should also be noted that in generous welfare states caregiving is mainly provided by professionals, while in less developed welfare states the family is expected to provide most of the required care [17]. It was recently reported, moreover, that the health consequences of caregiving vary not only across different welfare regimes but even between countries having similar welfare state types [10]. Policies to support informal caregivers in Europe vary to a large degree, while studies of the effects of these policies and the support services they provide are rare [18].

The sociocultural context of the countries is also related to the fact that across the globe, caregivers are predominantly women [19–22], and this may be an important source of health inequalities. Nevertheless, men are assuming increasingly important roles as caregivers due to changes in population demographics [20]. Gender differences in relation to caregiver burden and health have been observed [23–25], but whether gender specific services are necessary remains undetermined [26]. Thus, the need to incorporate gender in studies about caregivers has been emphasized [23,27].

Within this conceptual framework, and assuming that the policies of support to caregivers seek to ameliorate the diverse determinants of their stress (contextual factors, moderators, stressors, and their burden and quality of life), we hypothesized that the current policies of support to caregivers in Europe would have an association with their health. We also hypothesized that the association would differ according to the type of policy and the gender of the caregiver. Therefore, the aim of the current study was to assess the relationships between policies of support available to caregivers and the health of the caregivers, considering separately the support services based on financial help (paid leave, tax benefits, etc.) and the support based on training and other non-financial services. The study was also designed to detect the possible role of gender in the relationship between policies and the health of caregivers.

## Materials and methods

### Data source and study sample

Data from Wave 5 of the Survey of Health, Ageing and Retirement in Europe (SHARE, release 1.0.0 from March 31, 2015) were used in the current study [28]. SHARE is a cross-national and longitudinal European research project collecting data among people aged 50 years and over and their partners, regardless of their age. Data collection in Wave 5 was performed in 2013 in 15 of the 20 countries participating in SHARE at that time. The Ethics Council of the Max-Planck-Society for the Advancement of Science carefully reviewed the materials of the SHARE project and attested that the overall research project and its procedures, the measures to assure confidentiality and data privacy, and the information given to the participants agree with international ethical standards. The SHARE study consists of a computer assisted personal interview of 90 minutes, on average, that is conducted at the respondent’s household by trained interviewers. Questions cover a wide range of topics, including health related, economic and social support variables. Data are freely available to the research community (www.share-project.org).

The original SHARE Wave 5 sample consisted of 65,281 respondents. Participants from Italy, Estonia and Israel were excluded from the present analysis due to lack of information regarding the availability of the 10 support policies that were considered in this study [2]. The current analytical sample was thus comprised of 13,507 caregivers from the following 12 countries: Austria, Germany, Spain, France, Belgium, Czech Republic, Sweden, Netherlands, Denmark, Switzerland, Luxembourg, Slovenia. We considered a participant to be a caregiver if she or he answered positively to one of the following two questions: *“Let us now talk about help within your household*. *Is there someone living in this household whom you have helped regularly during the last twelve months with personal care*, *such as washing*, *getting out of bed*, *or dressing*? *(By regularly we mean daily or almost daily during at least three months*. *We do not want to capture help during short-term sickness of family members)”* or *“In the last twelve months*, *have you personally given personal care or practical household help to a family member living outside your household*, *a friend or neighbor*? *Please exclude looking after grandchildren”*

### Measures

Gender, age, education, employment, marital status and household size were recorded for each participant. Education was collected as the number of years studying using the International Standard Classification of Education-97 (ISCED-97) codes of UNESCO. We collapsed the codes into three groups: (i) Low education, which included participants with no education at all and those with ISCED-97 codes 1 and 2 (primary and lower secondary education). (ii) Medium education, which included participants with ISCED-97 codes 3 and 4, corresponding to secondary and post-secondary non-tertiary education. (iii) High education, which included participants with ISCED-97 codes 5 and 6, corresponding to first and second stages of tertiary education. Marital status was coded in two groups depending on whether the participant lived with a partner (either married or in a partnership) or not. Employment was also clustered in two groups, reflecting whether the participant was employed or not. Household size was recorded as the number of people living in the household of the participant.

We also took into account a range of health indicators. The number of self-reported comorbidities was recorded and it included the following conditions: 1) heart attack (including myocardial infarction or coronary thrombosis or any other heart problem such as congestive heart failure); 2) high blood pressure or hypertension; 3) high blood cholesterol; 4) stroke or cerebral vascular disease; 5) diabetes or high blood sugar; 6) chronic lung disease such as chronic bronchitis or emphysema; 7) cancer or malignant tumor, including leukemia or lymphoma, but excluding minor skin cancers; 8) stomach or duodenal ulcer, peptic ulcer; 9) Parkinson’s disease; 10) cataracts; 11) hip fracture, other fractures; 12) Alzheimer's disease, dementia, organic brain syndrome, senility or any other serious memory impairment; 13) other affective or emotional disorders, including anxiety, nervous or psychiatric problems; and 14) rheumatoid arthritis, osteoarthritis, or other rheumatism. The number of times the participant saw/talked to a doctor, and the number of nights spent as an inpatient in a hospital in the last 12 months were also recorded. Finally, a self-reported measure of health was also considered, with one's health ranked as being excellent, very good, good, fair or poor.

### Characteristics of the countries and policies of support to caregivers

The countries were classified as being family-based or service based countries, using a classification previously used by other authors [10, 29]. This classification is based on the welfare model of each country, taking into consideration the social and cultural role of the families as informal caregivers, and the level of professional services used. Family-based countries are those with a strong role of family as the main supplier of care. Service-based countries are countries where the state provides most of the care, and where professional support is widely offered [10].

The diverse support policies available were aggregated into two variables: “Financial support”, and “Training and other types of support.” The score on each variable was a count that ranged from 0 to 5, reflecting the availability of the key types of support. The "financial support" variable included: 1) caregiver allowance, 2) allowance for the person being cared for, 3) tax credit; 4) additional benefits *(country-specific specific special support policies such as tax deductions*, *pension credits*, *nursing fees…)*, and 5) paid leave. The “training and other types of support” variable included: 1) unpaid leave, 2) flexible work arrangements, 3) training/education, 4) respite care, and 5) counseling.

### Extent of caregiving

The extent of caregiving was defined as a numeric variable ranging from 1 to 3. Participants scoring 1 gave care outside their household only, that is, participants who answered “yes” to the first probe detailed earlier, *“(personal care or practical household help to someone living outside your household)* and answered “no” to the second probe, *“(regular personal care to someone within the household)*. Participants scoring 2 gave care only within their household, that is, participants who answered “no” to the first question described earlier, and “yes” to the second question. Participants scoring 3 were those who gave care both inside and outside their household (i.e., they answered “yes” to both questions).

### Statistical analysis

We first conducted a descriptive and univariate analysis of the study variables. Distributional assumptions of normality were analyzed with the Shapiro-Wilk test. Bivariate analyses were also conducted using Chi-square, Mann Whitney’s and Kruskal Wallis tests. Odd ratios for categorical variables and Cohen’s d for continuous variables were calculated.

**Fig 1:**
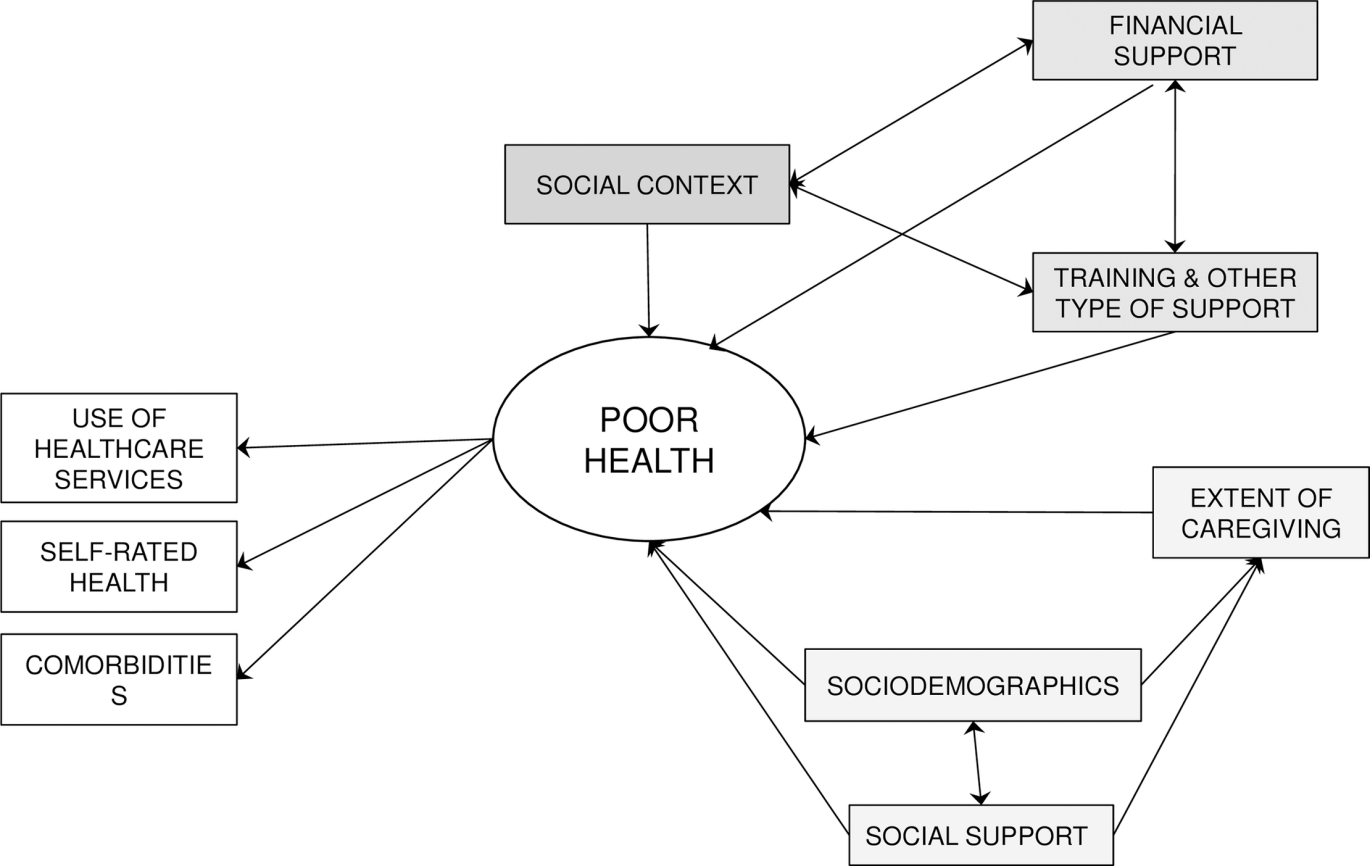
Theoretical model for poor caregiver’s health according to policies of support to caregivers.

A path analysis was subsequently performed according to the theoretical model shown in Fig 1, which was based on the Stress Process and Appraisal Models noted earlier [12,13]. Thus, we used the sociodemographic variables as contextual factors, and the social context-related variables and the policies of support as moderators. The stressor was the extent of caregiving. We used Maximum Likelihood as the estimator. Moreover, since the importance of possible gender-specific differences has been stressed [23,27], we performed this analysis three times. The first analysis included only caregiver men in the sample, the second included only caregiver women, and the third included the entire sample. The health of the respondents in our model was a latent variable made up of four indicators: 1) self-rated health, 2) the number of visits with a physician, 3) the number of comorbidities and 4) the number of nights spent in a hospital. Three indices were used to evaluate model fit: the comparative fit index (CFI), the Tucker-Lewis index (TLI), and the root mean square error of approximation (RMSEA). CFI and TLI values above 0.90 and RMSEA values of 0.06 or lower are considered indicators of an acceptable fit between the hypothesized model and the observed data [30]. The Chi square test, which is also generally used as a fit index, was not employed here. This is because in large samples such as ours, a significant chi square test is not meaningful [31].

Statistical tests were considered to be significant with a two-tailed p value <0.05. Processing and analysis of the data were performed using the statistical packages for windows IBM SPSS Statistics version 22.0.0.0 and the R SEM package version 3.1.9 [32,33].

## Results

### Characteristics of the sample

Our sample consisted of 13,507 caregivers from the 12 countries noted earlier. They constitute all the caregivers included in SHARE’s Wave 5 from these specific countries. There were 8,026 women in the sample (59.4%), and the mean age was 64.7 years (SD = 9.5). Each household had an average of 2.1 (SD = 0.9) members. Regarding education, the participants had studied 11.9 years on average (SD = 4.3). Only 3.6% (n = 488) of the sample were employed or self-employed. One third of the respondents reported having excellent/very good health (11.7% excellent and 21.2% very good), 30.7% reported good health, and the rest (29.3%) reported having fair/poor health. Participants had a visit or talked to a physician 6.8 times (SD = 9.7) during the last 12 months, and stayed in hospital 9.3 nights (SD = 15.7), on average. They had an average of 1.1 (SD = 1.2) long-term illnesses. Almost three out of four participants in our sample gave care to someone outside their household (75.7%; n = 10,222), one of five participants gave care to someone inside their household (19.0%; n = 2,568), and 5.3% (n = 717) gave care to persons inside and outside their household.

The characteristics of the sample by the extent of caregiving are shown in Table 1.

**Table 1:** Characteristics of the sample according to the extent of caregiving.

		Outside the household (n = 10,222)	Inside the household (n = 2,568)	Inside and outside the household (n = 717)
Gender, women, n (%)		6,099 (59.7)	1,488 (57.9)	439 (61.2)
Age, mean (SD)*		63.7 (8.8)	68.9 (10.8)	63.4 (9.2)
Education level, n (%)*				
	*Low*	2,649 (26.3)	1,209 (48.3)	220 (31.2)
	*Medium*	4,153 (41.2)	859 (34.3)	296 (41.9)
	*High*	3,286 (32.6)	435 (17.4)	190 (26.9)
Education years, mean (SD)*		12.3 (4.1)	10.3 (4.4)	11.9 (4.0)
Job, employed, n (%)*		0 (0.0)	358 (13.9)	130 (18.1)
Household size, mean (SD)*		2.0 (0.9)	2.4 (0.9)	2.5 (1.0)
Living in a family-based country, n(%)*		5,700 (55.8)	1,854 (72.2)	457 (63.7)
Times seen a physician, mean (SD)*		6.3 (9.1)	8.5 (11.5)	7.5 (11.0)
Total nights in a hospital, mean (SD)*		7.8 (11.3)	13.5 (23.1)	10.1 (20.4)
Num. chronic diseases, mean (SD)*		1.0 (1.1)	1.4 (1.3)	1.1 (1.1)
Self-reported health, n (%)*				
	*Excellent*	1,402 (13.7)	125 (4.9)	56 (7.8)
	*Very good*	2,409 (23.6)	324 (12.6)	132 (18.4)
	*Good*	3,915 (38.3)	888 (34.6)	285 (39.7)
	*Fair*	2,037 (19.9)	807 (31.4)	185 (25.8)
	*Poor*	455 (4.5)	422 (16.4)	59 (8.2)

* p<0.001

From a gender perspective (not shown in table), there were no differences between men and women regarding the extent of caregiving, being employed or unemployed, the number of long-term illnesses or the number of nights stayed in a hospital. Women, however, were one year younger than the men, on average, (64.3 vs 65.3 years; Mann-Whitney’s U = 20,557,058.50; p<0.001; Cohen’s d = 0.11) and had one year less of education (11.6 vs 12.3 years; Mann-Whitney’s U = 18,603,286.50; p<0.001; Cohen’s d = 0.16). They also saw a physician more often (7.0 vs. 6.5 times; Mann-Whitney’s U = 20,465,156.0; p<0.001; Cohen’s d = 0.05) and had fewer chronic diseases (1.0 vs. 1.2; Mann-Whitney’s U = 20,654,719.0; p<0.001; Cohen’s d = 0.09), although in all the cases, these differences had a small effect size (Cohen’s d smaller than 0.2). The OR (odds ratio) of being a female caregiver in a family-based country was 1.2 (95% CI = 1.1–1.2) compared to service-based countries, which, although significant, has a small effect size.

### Characteristics of the countries included in this study

In the sample there were 8,011 participants (59.3%) who lived in a family-based care country, while 5,496 (40.7%) resided in a service-based country. Among the financial support policies available, 5,750 (42.6%) participants lived in a country in which there is a caregiver allowance; 10,957 (81.1%) in a country with an allowance for the person being cared for; 3,673 (27.2%) in a country with a tax credit; 9,236 (68.4%) in a country with additional benefits for caregivers; and 8,270 (61.2%) in a country where paid leave is available. Regarding policies focused on training and other types of support, 6,708 (49.7%) participants lived in a country that provides unpaid leave; 7,014 (51.9%) in a country with flexible work arrangements; 11,966 (88.6%) in a country where education and/or training is provided to caregivers; and 13,110 (97.1%) in a country in which respite care is possible. Finally, counseling was available in all countries.

Table 2 shows the characteristics of the countries included in this study by the policies of support available to caregivers. In this table, the countries were ranked according to the number of policies of support available in each of the two categories: 1) financial support and 2) training and other type of support policies.

**Table 2:** Characteristics of the countries classified as family-based or service-based, and ranked according to the number of support policies available in two domains: financial support policies, and training and other type of support policies.

		Financial Support^A^	Training and other type of support^B^
Family-based care countries	Austria (n = 958)	2 ^a^^,^^e^	3 ^h^^,^^i^^,^^j^
	Germany (n = 1,541)	3 ^b^^,^^c^^,^^d^	3 ^f^^,^^g^^,^^i^
	Spain (n = 997)	3 ^b^^,^^d^^,^^e^	4 ^f^^,^^h^^,^^i^^,^^j^
	France (n = 1094)	4 ^b^^,^^c^^,^^d^^,^^e^	5 ^f^^,^^g^^,^^h^^,^^i^^,^^j^
	Belgium (n = 1721)	3 ^a^^,^^b^^,^^e^	5 ^f^^,^^g^^,^^h^^,^^i^^,^^j^
	Czech Rep. (n = 1700)	2 ^b^^,^^d^	4 ^g^^,^^h^^,^^i^^,^^j^
Service-based care countries	Sweden (n = 1378)	4 ^a^^,^^b^^,^^d^^,^^e^	3 ^h^^,^^i^^,^^j^
	Netherlands (n = 1171)	4 ^a^^,^^b^^,^^d^^,^^e^	5 ^f^^,^^g^^,^^h^^,^^i^^,^^j^
	Denmark (n = 1480)	2 ^a^^,^^e^	2 ^h^^,^^i^
	Switzerland (n = 641)	1 ^c^	3 ^h^^,^^i^^,^^j^
	Luxembourg (n = 397)	3 ^b^^,^^c^^,^^d^	3 ^f^^,^^h^^,^^j^
	Slovenia (n = 429)	1 ^e^	3 ^h^^,^^i^^,^^j^

Lowercase superscript letters indicate which are the specific support policies available in each country

^A^Financial support includes

^a^Carers allowance

^b^Allowance for the person being cared for

^c^Tax credit

^d^Additional benefits

^e^Paid leave.

^B^Training and other type of support includes

^f^Unpaid leave

^g^Flexible work arrangements

^h^Training / Education

^i^Respite care

^j^Counseling.

### Path analysis

Three models were performed in order to analyze a possible effect of the gender of the participants. The estimator values and the fit indexes did not vary according to gender (models 1 and 2). Consequently, we present here only the path analysis that was performed with the whole sample, including the gender variable as a covariate. The model queried the relationship of the respective study variables to the health of the caregiver respondents.

**Fig 2:**
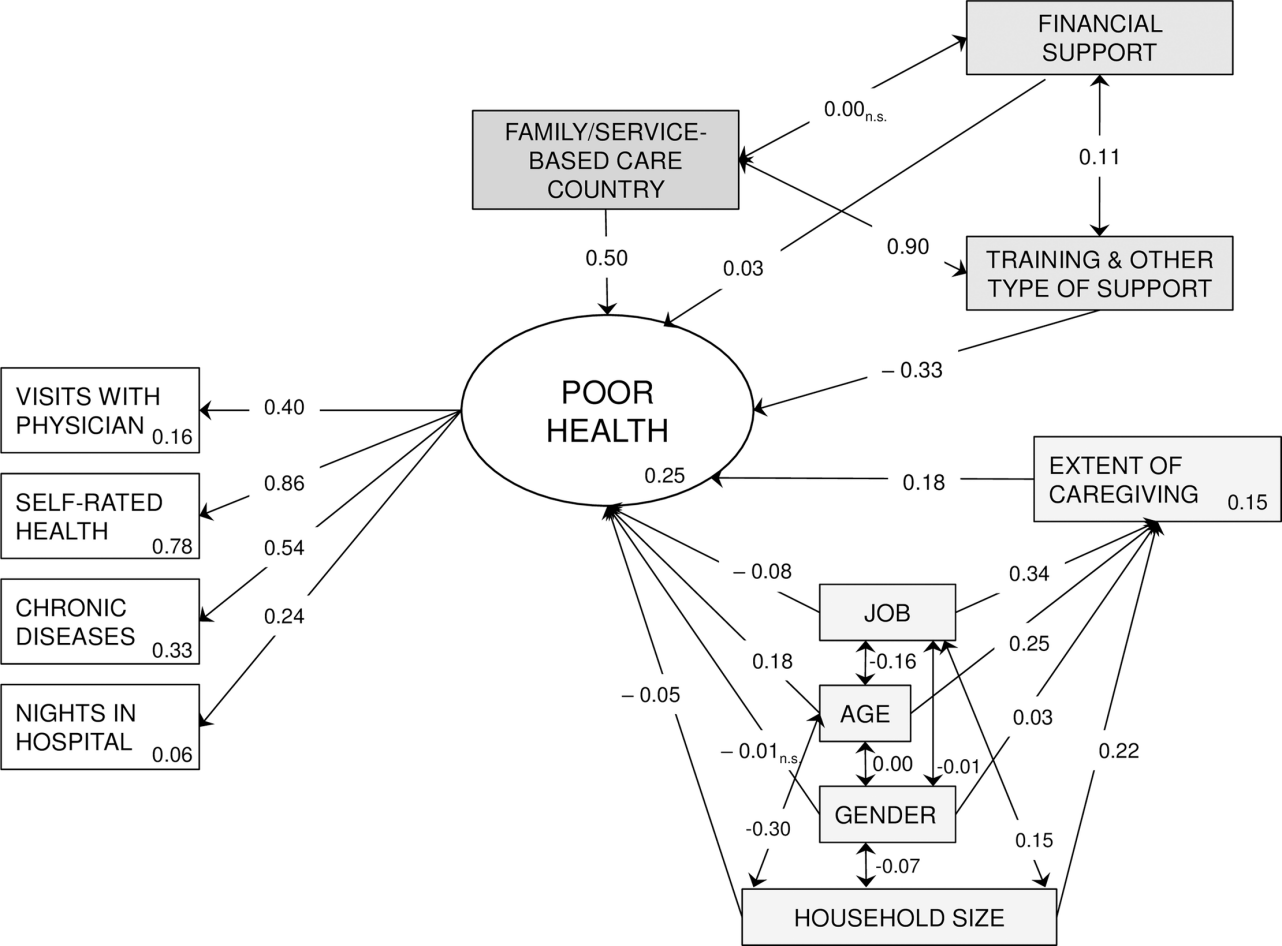
Association of caregiver’s health with the diverse policies of support to caregivers. All paths were significant except those marked as n.s. = non-significant, p≥0.05; Values within the boxes are r^2^ values.

The path analysis showed a good fit to the data with the following values for the fit indices: RMSEA  =  0.038 (90% CI = 0.035–0.040); CFI  = 0.975; TLI  = 0.950. Poor health of the caregivers was associated with living in a family-based care country (β = 0.50; 95% CI = 0.42–0.59), and with an increased extent of caregiving (β = 0.18; 95% CI = 0.15–0.22). Financial support policies had very little association with the health of the caregivers (β = 0.03; 95% CI = 0.01–0.04), but training and other type of support policies were positively associated (β = –0.33; 95% CI = –0.38 - –0.28). The complete results of this path model are presented in Fig 2.

## Discussion

Currently, informal caregivers are one of the most important assets to ensure care provision to the growing proportion of the population in need of care [3]. Thus, the policies of support to caregivers are of utmost importance to preserve their health, improve their well-being and guarantee they accomplish their function [11]. In this study, we used cross-national data from the SHARE survey to test our hypotheses that the current policies of support to caregivers in Europe have an association with the health of caregivers, and that this association varies according to the type of policy.

Our model explains 25% of the variance in the health of the caregivers, and the results are in agreement with previous research according to the main correlates studied so far [34]. Our results are in accordance with the formulated hypotheses and reflect the tenets of the Stress Process and the Appraisal models [12,13], which maintain that the policies of support to caregivers moderate the health of caregivers. In the current study, training and other type of support (including unpaid leave, flexible work arrangements, training and or education, respite care and counseling) were shown to have a strong positive association with the health of caregivers, while financial support to caregivers had almost no association.

Most financial support measures are earmarked for the cared-for person and not to the caregiver [18], which may explain the little effect these measures have in our study. For instance, a caregiver allowance is only available in four of the 12 countries included in our study, and the amount is generally low or is available for a limited period of time [18]. Training and other type of support measures, on the other hand, are earmarked for the caregiver. They mainly reduce the burden of caregiving by providing the caregivers with more time (i.e. respite care, flexible work arrangements or unpaid leave), which they can use for other activities or for their own care [8,11]. Other measures of support (i.e. training/education, counseling) have also been seen to help the caregivers to deal emotionally with their situation, and to give them skills to deal with care situations while improving the quality of the care they provide [35]. A systematic literature review revealed that informal caregivers of cohabiting elderly persons wish to have access to respite care, and to have contact with other people either for social or for learning purposes [36], which supports our results regarding these types of measures as those possibly having a larger effect on caregivers' self-reported health.

In the bivariate analyses, we saw that the extent of caregiving was not associated with the health status reported by the caregiver. Instead, it was associated with the care setting. Hence, the poorest health was reported by those giving care inside the household, while caregivers outside the household reported better health. This may be caused by the fact that being emotionally close to the recipient of care affects the caregiver’s health and because caregivers inside the household cannot evade the care situation easily [37]. It may also explain why caregivers inside and outside the household have better health than those who give care only inside the household. In agreement with this, other authors recently reported the poorest health in caregivers inside the household regardless of the welfare state [10]. However, the same authors analyzed intra-individual changes in a longitudinal study, and then almost no significant association was detected between caregiving inside the household and health [10]. In our case, those giving care inside the household only are older than those caregiving outside (either inside and outside or only outside). Thus, it could be that individuals with worse health choose (or can only) give care inside their household, and that caregiving outside one’s household is dependent on being younger and having better health [10].

According to our results, living in a family-based care country has a significant effect on the health of the caregivers. According to the “informal care model”, attitudes and beliefs towards caregiving, family, social network and the community, together with policy decisions and changes within society, are factors that lead a person to become an informal caregiver [4]. Recent research also suggests that women in the Southern welfare states of Europe (mostly family-based) suffer more from caregiving than those in the Northern states [38]. Living in a family-based care country may lead many people–who may already have bad health–to provide care to relatives just because this is the role they are expected to play. Appropriate support measures should be provided to all caregivers, but those with poorer health, mainly women, living in family-based care countries, may constitute a high risk-profile to consider separately for specific help policies.

Regarding gender specific results, as in most of the studies there were more women than men caregivers in our sample [19–21]. However, only minor gender-specific differences were detected in the bivariate analyses, and they all had small effect size. In the path analyses no gender-specific differences were seen, indicating that our results have the same validity for men and for women.

A number of limitations should be taken into account when interpreting our results: First, the availability of support measures in a country does not imply that the caregivers use them. In fact, studies have demonstrated that many caregivers fail to access the available support services [26,39]. Second, this is a cross-sectional study, which prevents providing cause-effect associations. Third, we have given the same weight to the diverse support measures, but some of them may have a larger effect on the caregivers’ health than others. Further research to elucidate this point should be performed. Fourth, social support has been seen to be an essential factor between caregiving and caregivers’ health. Since information on social support in our sample was limited, we used the household size as a partial indicator for social network. This same strategy has been employed previously by other authors using the SHARE dataset [6] and other datasets [40]. Fifth, this study has data only on persons aged 50 years old and over. While this age group constitutes the majority of caregivers, it should be noted that the results of our analysis cannot be extrapolated to younger providers of care.

### Conclusion

In sum, our study shows that non-financial support measures (education, training, respite care, non-paid leave and counseling) seem to have a larger protective impact on the health of caregivers than do financial support measures available in some European countries, regardless of the gender of the caregiver. There is a possibility that support policies to caregivers have an indirect effect on the care recipient’s health, which remains to be studied. From the caregivers’ perspective, our results may be of help in the development of new cost-effective policies supporting them in Europe. Future longitudinal studies using the SHARE dataset will allow establishing causal effects of the diverse available policies on the health of caregivers, and comparing these results between the countries included in this survey.
